# Consuming a Protein and Fiber-Based Supplement Preload Promotes Weight Loss and Alters Metabolic Markers in Overweight Adults in a 12-Week, Randomized, Double-Blind, Placebo-Controlled Trial

**DOI:** 10.1093/jn/nxac038

**Published:** 2022-02-25

**Authors:** Erin L Glynn, Stephen A Fleming, Caitlyn G Edwards, Michael J Wilson, Malkanthi Evans, Heather J Leidy

**Affiliations:** Scientific Affairs, Beachbody, LLC, El Segundo, CA, USA; Research and Development, Beachbody, LLC, El Segundo, CA, USA; Traverse Science, Inc., Champaign, IL, USA; Traverse Science, Inc., Champaign, IL, USA; Research and Development, Beachbody, LLC, El Segundo, CA, USA; KGK Science, Inc., London, Onatario, Canada; Department of Nutritional Sciences, University of Texas at Austin, Austin, TX, USA

**Keywords:** protein, fiber, nutrition, body composition, DXA, metabolic health, adiponectin

## Abstract

**Background:**

Higher protein and fiber diets promote weight management and metabolic health.

**Objectives:**

This study aimed to determine if greater weight loss and positive changes in metabolic outcomes could be achieved with twice-daily consumption of a high-protein and fiber-based multi-ingredient nutritional shake (HPF) compared with an isocaloric low-protein, lower fiber-based placebo (LPF).

**Methods:**

Study procedures were conducted by an independent research organization under clinicaltrials.gov registration NCT03057873. Healthy overweight and obese adults [*n *= 206; BMI (kg/m^2^): 27–35; 70% female] were randomly assigned to HPF or LPF. All participants were prescribed an energy-restricted diet (500 kcal/d less than energy needs) and consumed a HPF (17 g protein, 6 g fiber) or LPF (1 g protein, 3 g fiber) shake 30 min before breakfast and lunch for 12 wk. Primary outcomes included body weight and total body fat percentage. Blood samples were collected at days (D) 0, 28, 56, and 84 for secondary analyses related to metabolic markers of health.

**Results:**

Although weight loss occurred in both groups, HPF had greater weight loss at D84 compared with LPF (–3.3 kg vs. –1.8 kg, *P* < 0.05). Percentage body fat decreased in both groups (HPF: –1.33%, LPF: –1.09%; *P* < 0.001) with no differences between groups. Serum total cholesterol, LDL cholesterol, and oxidized LDL decreased between –5.1% to –8.3%, whereas adiponectin increased over time in both groups; these changes occurred to a greater extent in HPF compared with LPF (all *P* < 0.05).

**Conclusions:**

A multi-ingredient HPF nutritional supplement shake consumed as a preload before breakfast and lunch positively influenced weight management and metabolic outcomes in overweight adults compared with an LPF placebo. These findings suggest that specific nutrient factors (i.e., potentially including protein, fiber, and bioactive content) other than calorie reduction alone influence the success of a weight-loss regimen. This trial was registered at www.clinicaltrials.gov as NCT03057873,

## Introduction

Obesity is a global epidemic that is estimated to contribute to over 4 million deaths every year (1). In the United States alone, billions are spent annually on obesity-related health complications, including diabetes and dyslipidemia (2). Currently, lifestyle modifications, pharmaceutical therapies, and surgical interventions serve as the principal methods of obesity treatment. Lifestyle modifications include reductions in caloric intake and increases in physical activity. These approaches remain the most practical and widespread methods to controlling weight; however, long-term adherence remains a challenge, particularly among overweight and obese adults (3).

One of the challenges of reduced-calorie diets is the inability to control appetite. Reductions in food intake can lead to the activation of neurological pathways that increase hunger and food cravings (4). Developing dietary interventions that take into consideration these signaling pathways may be important in helping reduce daily food intake to sustain caloric restriction. Adjusting nutrient intake has the potential to serve as an effective strategy for increasing feelings of satiety, which can lead to improved appetite control. Diets composed of either high protein or high fiber have been proposed as sustainable dietary strategies for weight loss, prevention of weight regain following loss, and management of obesity-related comorbidities, due in part to their associations with satiety (5–8).

High-protein, energy-restricted diets containing between 1.2 and 1.6 g protein · kg^–1^ · d^–1^ (∼25–35% of daily intake as protein) have led to greater weight and fat loss, and greater preservation of lean mass, compared with lower protein diets (5). In addition, improvements in satiety, glycemic control, and cardiometabolic risk factors have been reported with higher protein diets (5). Given that the protein quantities within such studies are twice what Americans consume (9), others have explored whether similar improvements exist at lower quantities. Observational and experimental evidence suggests that a single high-protein meal is sufficient to elicit improvements in satiety, glycemic control, and body composition (10–13). However, within-meal protein quantities are still quite high, ranging from 30 to 45 g protein/meal. These quantities reduce generalizability and introduce difficulty in making protein recommendations that can be followed by the general population.

With regard to increased dietary fiber, a recent systematic review and meta-analysis reported greater reductions in BMI, body weight, and body fat, and greater improvements in cardiometabolic outcomes with the consumption of soluble fiber compared with placebo (14). However, similar to protein, the fiber quantity eliciting these improvements was fairly high (30 g). Another review reported that during ad libitum energy intake, an average (mean) of an additional 14 g fiber/d for >2 d is associated with a 10% decrease in energy intake and subsequent weight loss (15). Despite the well-defined benefits of fiber consumption, less than 3% of Americans consume the Adequate Intake recommendations for dietary fiber intake (16).

Recent focus has shifted towards examining whether a combination of more moderate protein and fiber intake may elicit similar beneficial effects shown at higher amounts of protein and fiber consumption. For example, Douglas et al. (17) compared the consumption of a single high-protein (34 g)/low-fiber (1 g) meal with that of a more moderate-protein (14 g)/fiber (5 g) meal in healthy adults. Similar appetite and satiety responses, as well as similar daily food intakes, were observed when replacing some protein (∼20 g) with fiber (+4 g). Thus, while limited data exist compared with very high protein intake, the combination of more moderate protein with fiber may have a synergistic effect on appetite, satiety, and/or subsequent food intake.

We have previously demonstrated that the acute consumption of the high-protein/fiber supplement shake (HPF) preload used in the current study (17 g protein, 6 g fiber) resulted in greater reductions in the desire to eat and hunger compared with the same low-protein/lower-fiber placebo (LPF) of this study (1 g protein, 3 g fiber) (18). Bonnema et al. (19) examined the effects of consuming breakfasts varying in protein and fiber on satiety, glycemic control, and food intake in a short-term study (participants were tested the same day on which consumption occurred). The more moderate protein (20 g)/fiber (7 g) meal led to similar reductions in postprandial glucose and subsequent food intake compared with the high-protein (30 g)/low-fiber (1 g) breakfast, and both reduced glycemic response and food intake compared with a lower-protein (10 g)/low-fiber (1 g) breakfast (19). While these studies support the consumption of meals containing a combination of more moderate protein and fiber over the short term, whether these improvements elicit long-term changes in weight loss or body composition has yet to be explored. Therefore, this study aimed to determine if greater weight loss and greater changes in body composition and metabolic outcomes could be achieved following a 12-wk energy-restricted diet that included twice-daily consumption of a protein and fiber-based multi-ingredient nutritional supplement shake (HPF) compared with an isocaloric LPF in adults with overweight and obesity. Secondary outcomes included metabolic parameters, glycemic control, and adipokine concentrations. Last, given that energy restriction in combination with changes in dietary patterns might alter mood and attitudes towards food, exploratory outcomes related to ingestive behavior and well-being were also included.

## Methods

### Participants

Two hundred and six healthy adults (**Table 1**) were recruited from 13 February 2017 to 24 July 2018 from the communities of London, Ontario, Canada; Toronto, Ontario, Canada; Orlando, Florida, and Chicago, Illinois. Screening included questionnaires of medical history, concomitant therapies including dietary supplement use, assessments of vital signs, height and weight, a pregnancy test (females of child-bearing potential only), and a fasted venous blood draw. Subsequently, enrolled participants were between the ages of 25 and 50 y, had a BMI (in kg/m^2^) of 27.0–35.0, and demonstrated maintenance of a stable weight for the past 6 mo (as defined as not having gained or lost >5 kg of body weight). Exclusion criteria included pregnant or breastfeeding females, smokers, and medical history of hypercholesterolemia, diabetes (type 1 and 2), hypertension, and/or eating disorders, among many others. Full inclusion and exclusion criteria and criteria changes during the study may be found in the **Supplemental Methods**.

**TABLE 1:** Baseline characteristics of overweight adults who consumed supplement shakes differing in protein and fiber for 12 wk^1^

	LPF		HPF	
	ITT (*n* = 103)	PP (*n* = 68)	ITT (*n* = 103)	PP (*n* = 65)
Women, %	68.9	73.5	71.8	72.3
Age, y	36.1 ± 7.7	36.3 ± 1.0	37.9 ± 7.9	38.6 ± 1.0
Weight, kg	84.8 ± 11	83.5 ± 1.4	87.1 ± 11	88.4 ± 1.4
BMI, kg/m²	30.4 ± 0.2	30.2 ± 0.2	30.6 ± 0.2	30.6 ± 0.3
Body fat, %	43.4 ± 7.8	44.1 ± 1.0	42.8 ± 8.4	42.7 ± 1.0
Waist circumference, cm	98.7 ± 8.4	98.7 ± 1.0	98.0 ± 7.9	98.4 ± 1.0

1 Values are unadjusted arithmetic means ± SEMs unless otherwise stated. HPF, group provided a high-protein, high-fiber supplement shake; ITT, intent-to-treat analysis; LPF, group provided a low-protein, lower-fiber placebo supplement shake; PP, per-protocol analysis.

### Experimental design

This was a 12-wk (84-d), randomized, double-blind, placebo-controlled, multisite, parallel-arm trial (clinicaltrials.gov, NCT03057873) with independent data collection (KGK Science, London, ON, Canada). This study was reviewed and approved by the Natural Health Product Directorate, Health Canada, and a research ethics board (Institutional Review Board Services, Aurora, ON, Canada), and conducted in compliance with ICH (The International Council for Harmonisation of Technical Requirements for Pharmaceuticals for Human Use) Guidelines for Good Clinical Practice. All participants provided written informed consent prior to study participation.

Following enrollment, participants completed a 7-d run-in period. Seven days prior to their baseline [day (D) 0] visit, a qualified nutritionist provided instructions on completing the 3-d food record, Bowel Habits Diary (BHD), and Stanford 7-Day Physical Activity Recall Questionnaire. The BHD was completed on days participants filled out the 3-d food record throughout the study. Following the run-in period, eligible participants were randomly assigned to 1 of 2 treatment arms by a blinded member of the research team on study D0 (baseline).

**FIGURE 1:**
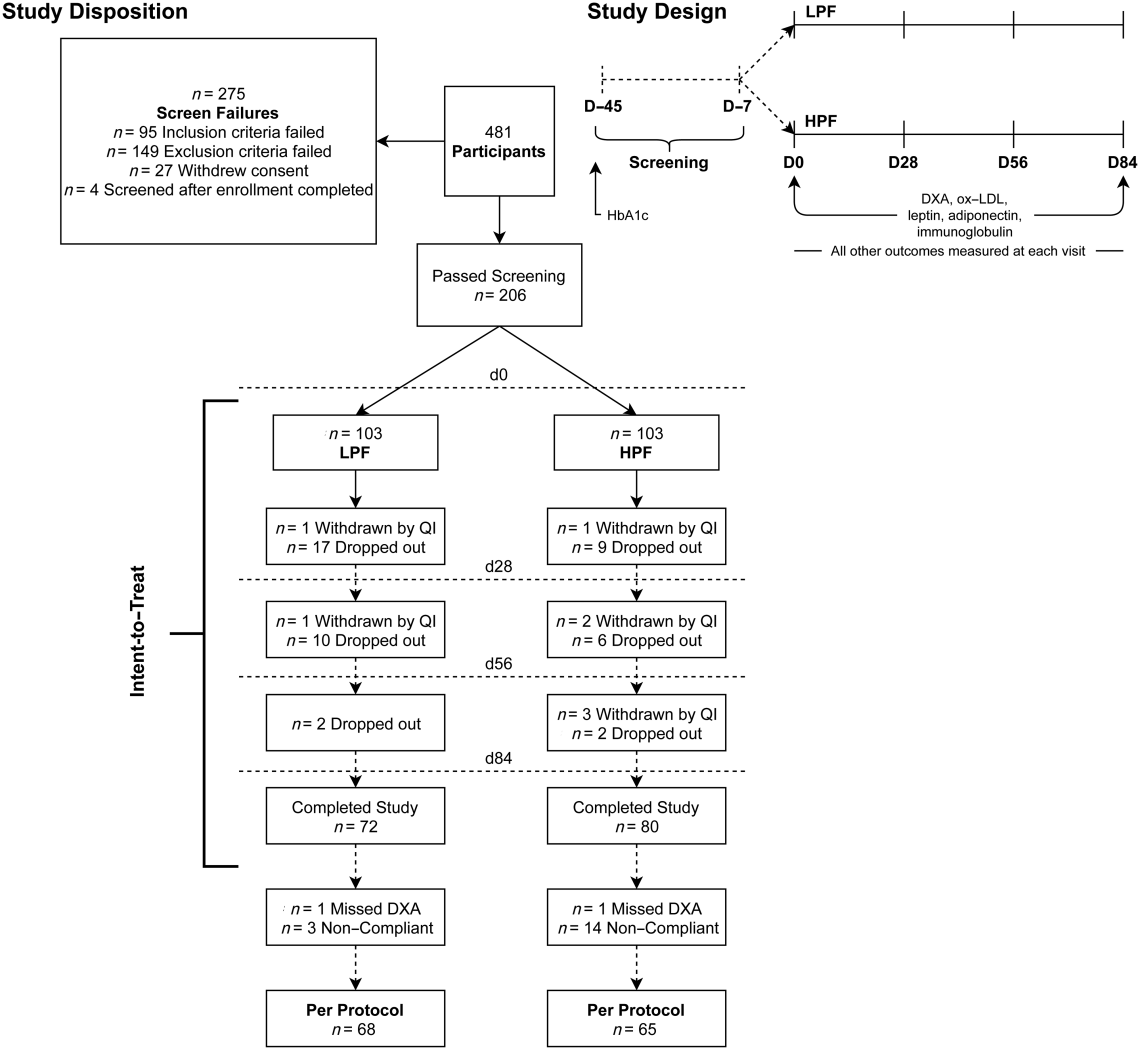
Study disposition and flow of an independently conducted, randomized, double-blind, placebo-controlled, multicenter clinical trial on overweight adults who consumed supplement shake preloads with low protein and lower fiber (LPF) or high protein and high fiber (HPF) for 12 wk. Participants were screened on D45 and D7, after which they were randomly assigned to each treatment. D/d, day; HbA1c, glycated hemoglobin; ox-LDL, oxidized LDL; QI, qualified investigator.

Participants were assigned a randomization code from a list generated by www.randomization.com and allocated to intervention groups in a 1:1 ratio. Outcomes were measured on D0, 28, 56, and 84. Participants arrived at each testing visit following a 10–12-h overnight fast. At each visit, the following measurements were taken: body weight, heart rate, blood pressure, and waist and hip circumference, and a venous blood draw was collected (**Figure 1**). Blood glycated hemoglobin (HbA1c) was assessed at screening and at D84. Body composition was assessed within a ±3-d window of D0 and D84. Questionnaires assessing physical activity (Stanford 7-Day Physical Activity Recall; data not shown), mood (Profile of Mood States-2), binge eating tendencies (Binge Eating Scale), and gastrointestinal symptoms (Modified Gastrointestinal Symptom Rating Scale), and the BHD with Bristol stool scales were completed at or between each visit and reported in the Supplemental Methods.

### Dietary intervention and counseling

The diet plan was based on the Diabetic Exchange List (Exchange Diet) as the basis of a meal planning system originally designed by a committee of the American Diabetes Association and the American Dietetic Association. Both groups were assigned a 500-kcal/d deficit from calculated energy needs via an exchange-based diet plan and were advised on guidelines for physical activity (2.5 h/wk of moderate to vigorous-intensity exercise). Predetermined numbers of exchanges in the 6 food groups were assigned based on caloric needs in 100-kcal increments to achieve approximately 15% of daily energy from protein, 55% from carbohydrate, and 30% from fat. Caloric needs were calculated as the mean of the Mifflin-St. Jeor and Katch-McArdle formulas, multiplied by an activity factor of 1.3. From the assigned kcal, 320 kcal were designated for the assigned treatment supplement, and the rest could be freely allocated based on the exchange-based calorie-restricted diet plan. The macronutrient breakdown of the food portion of the calorie-restricted diet (not including the supplement shakes) aligned with the Acceptable Macronutrient Distribution Ranges (AMDRs) and was 15% of daily energy from protein, 55% from carbohydrate, and 30% from fat to be distributed over 3 meals and 2 snacks. At each study visit between D0 and D84, participants met with the nutritionist who reviewed their food and physical activity records since the previous visit. The participants were counselled to adhere to their assigned diet plan and the general physical activity recommendations based on the nutritionist's review of these records.

Participants in the HPF intervention group consumed a commercially available dietary supplement shake (Shakeology Chocolate flavor; provided by the study sponsor Beachbody LLC, Santa Monica, CA) containing 17 g protein and 6 g fiber. The dietary supplement shake was composed of a protein blend (whey, pea) and fruit-, vegetable-, and plant-derived powders, as well as vitamins, minerals, prebiotics, and probiotics. Fiber sources include cocoa, pea fiber, xanthan gum, inulin, flaxseed, chicory root, and chia seed powder. The LPF control group consumed a maltodextrin-based placebo supplement that contained 1 g protein and 3 g fiber. The LPF was matched for caloric content, color, flavor palatability, and vitamin and mineral fortification as in the HPF supplement. Product nutritional information is presented in **Table 2**, and full product ingredients are listed in the Supplemental Methods. Both groups were instructed to consume their respective shake preloads 30 min prior to both breakfast and lunch.

**TABLE 2:** Nutritional composition of supplement shakes^1^

	LPF	HPF
Serving size, g	42.6	42
Energy, kcal	160	160
Protein, g	1	17
Fat, g	0.5	2
Carbohydrate, g	37	17
Sugar, g	13	7
Fiber, g	3	6
Beverage volume, mL	295.7	295.7
Viscosity (prepared beverage), cP	568	450

1 cP, centipoise; HPF, high-protein, high-fiber supplement shake; LPF, low-protein, lower-fiber supplement shake.

### Dietary intake and adherence

Prior to D0 and subsequently every 2 wk during the study, participants completed 3-d food records (2 weekdays and 1 weekend) that were returned at testing visits. The means from these records were used for further analyses. Trained nutritionists on the research study staff provided guidance on completing the diet records and analyzed records. Product compliance was assessed by counting the amount of returned unused product at each testing appointment. Noncompliance was defined as participants consuming <80% or ≥120% of product provided. The percentage of product compliance was calculated by determining the number of dosage units divided by the number of dosage units expected to have been consumed multiplied by 100. Food records demonstrating <900 kcal intake for that day were excluded from analysis, as these represented unlikely records of true intake since participants were assigned a minimum of 1200 kcal/d. There was no upper intake limit for the exclusion of food records. For estimation of dietary intake, intakes from foods and the shake preload were included in the analysis.

### Clinical assessment

A fasted venous blood draw was completed at all time points for assessment of metabolic health outcomes including lipids [total cholesterol, triglycerides, HDL cholesterol, LDL cholesterol, and oxidized LDL (ox-LDL; D0 and D84 only)], fasting glucose, insulin, leptin, adiponectin (leptin, adiponectin; D0 and D84 only); clinical blood chemistry; immune markers [IgG, IgA, IgM, complement (C) 3, C4; D0 and D84 only]; and a comprehensive metabolic panel. HbA1c was assessed at initial screening and D84. All analyses were conducted by Lab Corp/Life Labs (London, ON, Canada), with the exception of ox-LDL, which was analyzed by KGK Science, Inc. Whole blood was collected into EDTA-coated tubes for complete blood counts and HbA1c analysis. Serum was collected in serum separation tubes (SST) for lipid analyses, electrolytes, blood chemistry, liver enzymes, immune markers, leptin, adiponectin, insulin, and glucose.

### Body composition

Participant weight and height were taken in duplicate with shoes removed and bladder empty. BMI was calculated as weight (kg)/height (m^2^). For further assessment of body composition, DXA (Lunar Prodigy Advanced; GE Healthcare) was conducted within a ±3-d window of D0 and D84 by trained technicians. Participants were instructed to refrain from alcohol consumption for 48 h and exercise for 12 h and to fast for 6 h before completing their DXA scan. Body-composition assessments were measured in grams and converted to kilograms for analysis.

### Power calculations and planned analyses

Change in body weight and percentage body fat from D0 to D84 were the primary outcomes. A statistical analysis plan was created before data analysis. An intent-to-treat (ITT) analysis was independently conducted by KGK Science, Inc., with a follow-up per-protocol (PP) analysis conducted by Traverse Science, Inc., that only included compliant participants with complete data from the primary outcomes. In both cases, the analysis was performed in a blinded manner. Before study enrollment, a power analysis [using G*Power 3.1.9.4 (20)] using estimates from previous literature (21) identified the need for a sample size of 69 participants per group to detect a mean difference of 1.5 kg (3.1 kg SD) in body weight lost between 2 independent groups (an effect size of *d* = 0.48) assuming α = 0.05 and β = 0.20 using a 2-tailed independent *t* test. Assuming an attrition rate of 30%, approximately 200 participants in total would be needed to be enrolled in the study. Before the PP analysis, a sensitivity analysis was conducted to identify the effect size expected given the achieved sample size after the exclusion of noncompliant participants and those with incomplete data. The following parameters were assumed using the ANOVA repeated-measures module with a within-between interaction: α = 0.05, β = 0.20, *n* = 133, 2 groups (LPF and HPF), 4 measurements (D0, D28, D56, and D84), assumed correlation among measurements of 0.50, and nonsphericity correction of ε = 0.34–1. This achieved effect sizes of *f* = 0.10–0.15. Thus, it was determined that the study was adequately powered to identify even small effect sizes.

### Statistical modeling

Significance for all analyses was made at α = 0.05 unless otherwise specified.

#### ITT analysis

The ITT analysis was used for the primary outcomes only using SAS 9.3 (SAS Institute). To assess between-group changes, data were modeled using ANCOVA predictors including group (between-participants), time (within-participant), and interaction of group by time. For percentage body fat, the only predictor included was group, and effects of time were analyzed using a within-group Wilcoxon signed-rank test. Baseline or gender were included as covariates using stepwise regression and included if the effect of the covariate was *P* ≤ 0.15 (body weight: sex; % body fat: baseline value and sex). The covariance structure with the smallest Akaike's Information Criterion value was used [body weight: heterogenous AR(1); % body fat: diagonal]. Missing values were imputed using the Markov chain Monte Carlo method for making post hoc, between-group comparisons only. No imputation was performed when making post hoc, within-group comparisons.

#### PP analysis

Data from participants who met compliance criteria (consumed ≥80% and <120% of product provided) and had complete primary outcome data (D0 and D84 body weight and DXA scans) were included in the PP analysis. All tests were conducted using R software version 3.6.2 (R Foundation for Statistical Computing) (22). Raw values and change from baseline (for primary outcomes) were modeled. Sex and baseline value (i.e., the value of a given variable at D0) were considered covariates for all models, with the exception of dietary intake in which only sex was used as a covariate. Predictors included testing day as a fixed within-participants effect (time), group as a fixed between-participants effect, the interaction of time by group, and participant as a random variable. Data were modeled using generalized least-squares regression with a first-order autoregressive AR(1) covariance structure with homogenous variance. If a value was missing from a given time point, the observation was omitted from that point only. Outliers were not removed from analysis and imputation was not conducted. Post hoc mean comparisons were made using the emmeans package (23), and all data are presented using estimated marginal means, adjusted for sex and baseline value where appropriate. Significance for post hoc comparisons was determined at α = 0.05 using a Tukey adjustment.

## Results

### Study disposition and demographics

A total of 481 participants were screened for inclusion (Figure 1). Of these, 275 failed screening criteria, leaving 103 per group for inclusion. Due to drop-out and withdrawals throughout the study, 152 participants completed all study visits (LPF: 70% drop-out; HPF: 78% drop-out). According to the PP analysis, participants were omitted if they did not have complete primary outcome data (body weight or body fat %) or were noncompliant (consumed <80% or >120% of assigned intervention product during any 4-wk period). After applying these criteria, a total of 133 participants remained, with 68 and 65 in the LPF and HPF groups, respectively. Participants were a mean of 37.4 y old, primarily female (73%), and Western European White (62%) (Table 1).

### Compliance and dietary intake

Of participants who completed the trial, 3 of 72 participants were noncompliant in the LPF group (95% compliance) and 14 of 80 participants were noncompliant in the HPF group (83% compliance). Total energy intake decreased similarly between groups over time but only reached statistical significance in the LPF group compared with baseline (**Table 3**; *P* < 0.05). From D28 onward, the HPF group consumed more protein from baseline and the LPF group consumed less, with both groups differing from each other (*P* < 0.001; Table 3). Both groups increased energy intake from carbohydrates compared with baseline, with the LPF group consuming more energy from carbohydrates (Table 3; *P* < 0.001). Both groups exhibited a decrease in the intake of energy from fat, with the LPF group consuming the least energy from fat (Table 3; *P* < 0.001). At D0, the LPF group consumed 6.6 g/d more fiber than the HPF group (Table 3; *P* < 0.05). Fiber intake did not change over time in the LPF group, whereas intake of fiber increased in the HPF group and was more than in the LPF group on D56 and D84 (Table 3; *P* < 0.05). Total sugar intake was similar between groups at baseline, with the HPF group demonstrating a reduction in sugar intake from baseline that was lower than the LPF group from D28 onwards (Table 3; *P* < 0.05).

**TABLE 3:** Mean energy and nutrient intakes of overweight adults who consumed supplement shakes differing in protein and fiber for 12 wk^1^

Time point					*P* ^2^		
Variable and group	D0	D28	D56	D84	Time	Group	Time × group
Sample size, *n*							
LPF	66	61	65	63	—	—	—
HPF	65	61	62	61	—	—	—
Total energy, kcal/d							
LPF	2270 ± 130	1750^3^ ± 110	1700^3^ ± 100	1710^3^ ± 110	<0.001	0.390	0.747
HPF	2030 ± 130	1730 ± 110	1690 ± 110	1670 ± 110	—	—	—
Protein, % of energy/d							
LPF	18.1 ± 0.4	14.4^3^ ± 0.3	13.6^3^ ± 0.3	13.6^3^ ± 0.3	0.119	<0.001	<0.001
HPF	18.2 ± 0.4	21.0^3^^,^^4^ ± 0.4	21.2^3^^,^^4^ ± 0.3	21.7^3^^,^^4^ ± 0.3	—	—	—
Carbohydrate, % of energy/d							
LPF	48.5 ± 0.7	59.5^3^ ± 0.7	60.3^3^ ± 0.6	59.9^3^ ± 0.6	<0.001	<0.001	<0.001
HPF	47.3 ± 0.7	50.9^3^^,^^4^ ± 0.7	50.8^3^^,^^4^ ± 0.6	50.3^3^^,^^4^ ± 0.6	—	—	—
Fat, % of energy/d							
LPF	33.4 ± 0.7	26.2^3^ ± 0.6	26.1^3^ ± 0.6	26.5^3^ ± 0.6	<0.001	<0.001	0.903
HPF	34.5 ± 0.7	28.2^3^^,^^4^ ± 0.6	28.0^3^^,^^4^ ± 0.6	28.1^3^^,^^4^ ± 0.6	—	—	—
Fiber, g/d							
LPF	25.0 ± 2.3	23.2 ± 2.0	22.1 ± 1.9	22.4 ± 2.0	0.297	0.037	0.011
HPF	18.4^4^ ± 2.3	27.6^3^ ± 2.0	28.0^3^^,^^4^ ± 1.9	27.8^3^^,^^4^ ± 1.9	—	—	—
Sugar, g/d							
LPF	80.0 ± 3.2	78.3 ± 2.9	80.7 ± 2.7	79.1 ± 2.8	<0.001	<0.001	<0.001
HPF	85.6 ± 3.2	65.9^3^^,^^4^ ± 2.9	63.3^3^^,^^4^ ± 2.8	64.6^3^^,^^4^ ± 2.8	—	—	—

1 Values are estimated marginal means ± SEs. Intake data include dietary intake of the supplement shake. Total sample size was *n* = 68 (LPF) and *n* = 65 (HPF). D, day of trial; HPF, group provided a high-protein, high-fiber supplement shake; LPF, group provided a low-protein, lower-fiber supplement shake.

2 Data from the per-protocol analysis, where noncompliant participants were removed and data modeled using generalized least-squares regression with time as a within-participants fixed effect, group as a between-participants fixed effect, their interaction, and sex as a covariate.

3 Different from day 0, *P* < 0.05 (Tukey-adjusted within-participant comparison).

4 Different from LPF at that time, *P* < 0.05 (Tukey-adjusted between-group comparison).

### Primary outcomes

**FIGURE 2:**
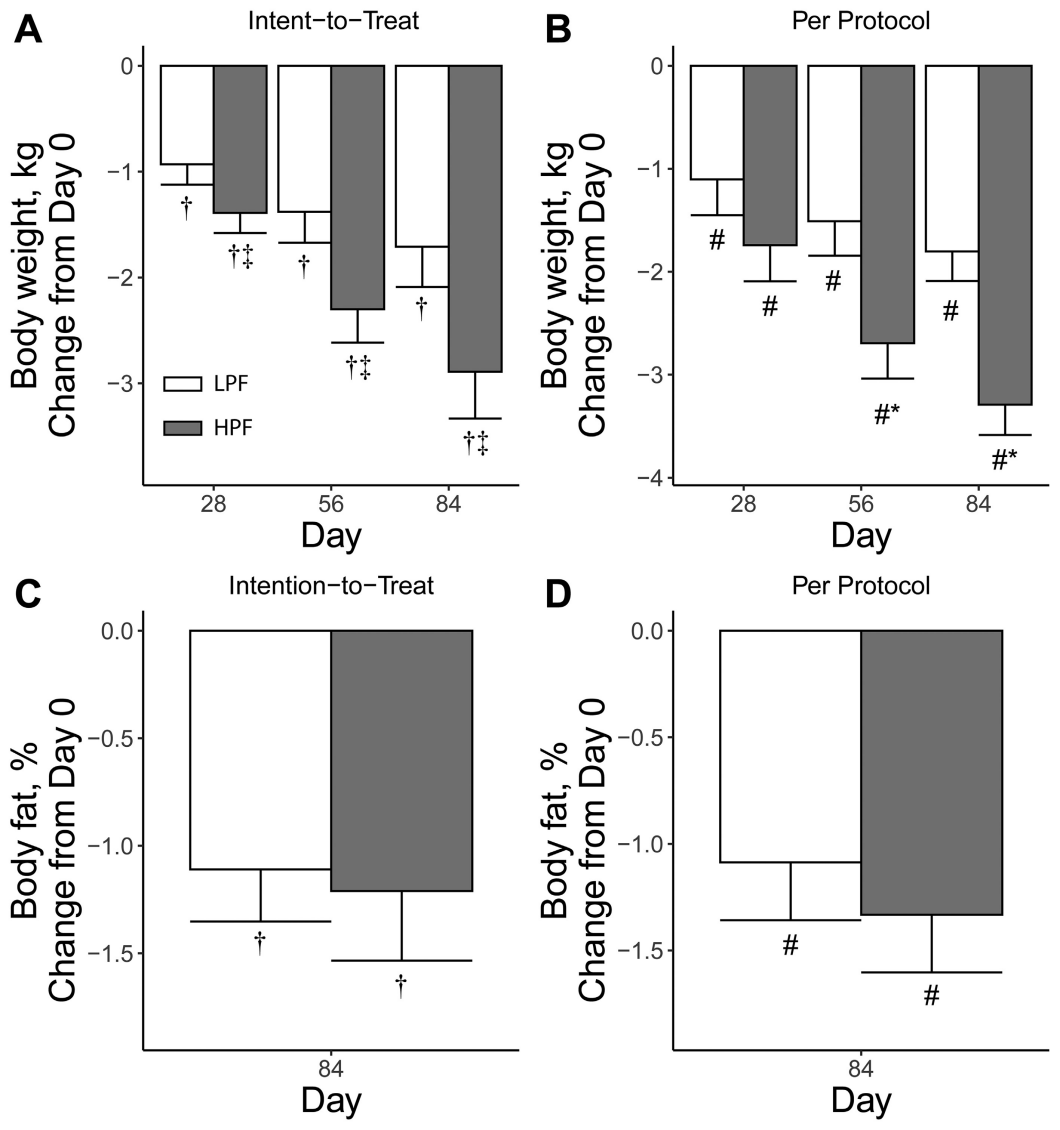
Body weight and body fat percentage of overweight adults who consumed supplement shake preloads with high protein and high fiber (HPF) or low protein and lower fiber (LPF) for 12 wk. Intent-to-treat analyses on the change from baseline (day 0) in body weight (A) and body fat % (C) and per-protocol analyses on the change from baseline (day 0) in body weight (B) and body fat % (D). For the intent-to-treat analysis, between-group differences were assessed using ANCOVA, with time as a within-participants fixed effect, group as a between-participants fixed effect, and their interaction [body-weight sample sizes: day 28, *n* = 89 (LPF) and *n* = 98 (HPF); day 56, *n* = 79 (LPF) and *n* = 89 (HPF); and day 84 *n* = 73 (LPF) and *n* = 80 (HPF); body-fat sample sizes, *n* = 71 (LPF) and *n* = 79 (HPF)]. For the per-protocol analysis, noncompliant participants were removed and data modeled using generalized least-squares regression with the same main and interaction effects, but with both baseline and sex as covariates [*n* = 68 (LPF) and *n* = 65 (HPF) for all time points]. Values are presented as estimated marginal means ± SEs. *Different from LPF at that time, *P* < 0.05 (Tukey-adjusted between-group comparison). ^#^Different from day 0, *P* < 0.05 (Tukey-adjusted within-participant comparison). ^†^Different from day 0, *P* < 0.05 (Wilcoxon signed-rank test). ^‡^Different from LPF at that time, *P* < 0.05 (Wilcoxon signed-rank test).

In both the ITT and PP analyses, both groups exhibited a decrease in body weight over time (ITT and PP, *P* < 0.001; **Table 4**), with participants in the HPF group exhibiting greater weight loss compared with baseline than those in the LPF group (ITT and PP, *P* < 0.05; **Figure 2**). In both the ITT and PP analyses, both groups exhibited a decrease in percentage body fat over time (within-group: ITT and PP, *P* < 0.05), and the HPF group did not differ from the LPF group at any time (ITT and PP, *P* ≥ 0.46). For change in body weight from baseline, the effect size (Cohen's *d*) between groups was 0.33 in the ITT and 0.63 in the PP analysis. Compared with no weight loss (average change in body weight of 0 kg), the effect size was 0.53–0.73 (ITT: LPF–HPF) or 0.76–1.39 (PP: LPF–HPF).

**TABLE 4:** Primary outcomes of overweight adults who consumed supplement shakes differing in protein and fiber for 12 wk^1^

	Time point				*P* ^2^		
Variable and group	D0	D28	D56	D84	Time	Group	Time × group
ITT analysis							
Weight, kg							
LPF	84.8 ± 1.1 (103)	83.1^3^ ± 1.2 (89)	82.5^3^ ± 1.3 (79)	82.5^3^ ± 1.4 (73)	<0.001	0.012	0.007
HPF	87.1 ± 1.1 (103)	85.6^3^^,^^4^ ± 1.2 (98)	84.9^3^^,^^4^ ± 1.2 (89)	84.9^3^^,^^4^ ± 1.2 (80)	—	—	—
Body fat, %							
LPF	44.4 ± 0.8 (103)	—	—	43.1^3^ ± 1.0 (71)	—	0.462	—
HPF	42.8 ± 0.8 (103)	—	—	41.8^3^ ± 1.0 (79)	—	—	—
PP analysis							
Weight, kg							
LPF	85.8 ± 0.3 (68)	84.7^5^ ± 0.3 (68)	84.3^5^ ± 0.3 (68)	84.0^5^ ± 0.3 (68)	<0.001	0.023	0.004
HPF	85.7 ± 0.3 (65)	84.0^5^ ± 0.3 (65)	83.0^5^^,^^6^ ± 0.3 (65)	82.4^5^^,^^6^ ± 0.3 (65)	—	—	—
Body fat, %							
LPF	43.2 ± 0.2 (68)	—	—	42.1^5^ ± 0.2 (68)	<0.001	0.531	0.572
HPF	43.2 ± 0.2 (65)	—	—	41.9^5^ ± 0.2 (65)	—	—	—

1 Values are estimated marginal means ± SEs, with sample size in parentheses. For all variables except for body weight and % body fat, only the results from the per-protocol analysis are described. D, day of trial; ITT, intent-to-treat; HPF, group provided a high-protein, high-fiber supplement shake; LPF, group provided a low-protein, lower-fiber supplement shake; PP, per-protocol.

2 For the ITT analysis, between-group differences were assessed using ANCOVA, with time as a within-participants fixed effect, group as a between-participants fixed effect, and their interaction. For the PP analysis, noncompliant participants were removed, and data modeled using generalized least-squares regression with the same main and interaction effects, but with both baseline value (taken at D0) and sex as covariates.

3 Different from day 0, *P* < 0.05 (Wilcoxon signed-rank test).

4 Different from LPF at that time, *P* < 0.05 (Wilcoxon signed-rank test).

5 Different from day 0, *P* < 0.05 (Tukey-adjusted within-participant comparison).

6 Different from LPF at that time, *P* < 0.05 (Tukey-adjusted between-group comparison).

### Secondary outcomes

Given that the results of the primary outcomes and conclusions did not differ between the ITT and PP analyses, the results for secondary outcomes are reported for the PP analysis only.

#### Body composition

At D84, the HPF group had a decrease in total lean tissue (*P* < 0.05), with no change in the LPF group (**Table 5**). However, when expressed as a percentage of total tissue, these differences were no longer apparent, and both groups had increased lean tissue percentage from baseline (*P* < 0.05). Both groups exhibited a reduction from baseline in waist and hip circumference (*P* < 0.05) starting on D28 (Table 5).

**TABLE 5:** Body composition of overweight adults who consumed supplement shakes differing in protein and fiber for 12 wk^1^

	Time point				*P* ^2^		
Variable and group	D0	D28	D56	D84	Time	Group	Time × group
BMI, kg/m²							
LPF	30.4 ± 0.1	30.0^3^ ± 0.1	29.9^3^ ± 0.1	29.8^3^ ± 0.1	<0.001	0.033	0.005
HPF	30.4 ± 0.1	29.8^3^ ± 0.1	29.5^3^^,^^4^ ± 0.1	29.3^3^^,^^4^ ± 0.1	—	—	—
Waist circumference, cm							
LPF	98.9 ± 0.5	96.3^3^ ± 0.6	95.7^3^ ± 0.6	95.1^3^ ± 0.5	<0.001	0.902	0.689
HPF	99.0 ± 0.5	96.7^3^ ± 0.6	95.5^3^ ± 0.6	94.6^3^ ± 0.6	—	—	—
Hip circumference, cm							
LPF	110 ± 0.4	109^3^ ± 0.5	108^3^ ± 0.5	107^3^ ± 0.4	<0.001	0.474	0.804
HPF	110 ± 0.4	109^3^ ± 0.5	108^3^ ± 0.5	107^3^ ± 0.5	—	—	—
Total tissue (DXA), kg							
LPF	82.7 ± 0.3	—	—	81.0^3^ ± 0.3	<0.001	0.028	0.016
HPF	82.8 ± 0.3	—	—	79.5^3^^,^^4^ ± 0.3	—	—	—
Total fat tissue, kg							
LPF	35.5 ± 0.3	—	—	34.0^3^ ± 0.3	<0.001	0.192	0.174
HPF	35.5 ± 0.3	—	—	33.2^3^ ± 0.3	—	—	—
Total lean tissue, kg							
LPF	47.3 ± 0.2	—	—	47.1 ± 0.2	<0.001	0.042	0.005
HPF	47.4 ± 0.2	—	—	46.4^3^^,^^4^ ± 0.2	—	—	—
Total lean tissue, %							
LPF	56.7 ± 0.2	—	—	57.8^3^ ± 0.2	<0.001	0.689	0.690
HPF	56.7 ± 0.2	—	—	58.0^3^ ± 0.2	—	—	—

1 Values are estimated marginal means ± SEs. Total sample size was *n* = 68 (LPF) and *n* = 65 (HPF). For all variables except for body weight and % body fat, only the results from the per-protocol analysis are described. D, day of trial; HPF, group provided a high-protein, high-fiber supplement shake; LPF, group provided a low-protein, lower-fiber supplement shake.

2 Data from the per-protocol analysis, where noncompliant participants were removed and data modeled using generalized least-squares regression with time as a within-participants fixed effect, group as a between-participants fixed effect, their interaction, and sex and baseline value (taken at D0) as a covariate.

3 Different from day 0, *P* < 0.05 (Tukey-adjusted within-participant comparison).

4 Different from LPF at that time, *P* < 0.05 (Tukey-adjusted between-group comparison).

#### Glycemic, cholesterol, and hormone profiling

Fasting insulin decreased over time for both groups (*P* < 0.05) and was generally lower in the HPF group than in the LPF group, but only statistically lower in the HPF group on D28 (*P* < 0.05; **Table 6**). Fasting blood glucose changed over time, although this was only decreased from baseline in the LPF group on D84 (*P* < 0.05). At D84, only the HPF group demonstrated a decrease in percentage HbA1c from baseline (*P* < 0.05). Both total cholesterol and LDL cholesterol changed over time and were lower in the HPF group but were only significantly less than baseline on D84 for the HPF group (*P* < 0.05). Although HDL cholesterol was lower from baseline on D28 for the HPF group (*P* < 0.05), there were no differences between groups in HDL cholesterol (*P* = 0.24). Triglycerides did not change with time (*P* = 0.57) but were lower in the HPF group (*P* < 0.05). Ox-LDL decreased with time (*P* < 0.05) but was only significantly lower from baseline in the HPF group (*P* < 0.05). Leptin concentrations were stable over time (*P* = 0.06), with no differences between groups (*P* = 0.87). Adiponectin increased over time (*P* < 0.05) and was greater in the HPF group (*P* < 0.05) but was only greater from baseline in the HPF group on D84 (*P* < 0.05).

**TABLE 6:** Metabolic parameters of overweight adults who consumed supplement shakes differing in protein and fiber for 12 wk^1^

	Time point				*P* ^2^		
Variable and group	D0	D28	D56	D84	Time	Group	Time × group
Fasting insulin, pmol/L							
LPF	80.5 ± 3.42	77.5 ± 3.45	80.0 ± 3.44	71.6 ± 3.43	0.001	0.025	0.401
HPF	79.5 ± 3.48	65.4^3^^,^^4^ ± 3.52	71.5 ± 3.51	66.7^3^ ± 3.49	—	—	—
Glucose, mmol/L							
LPF	5.02 ± 0.04	4.99 ± 0.04	5.01 ± 0.04	4.90^3^ ± 0.04	0.006	0.466	0.544
HPF	5.00 ± 0.04	4.99 ± 0.04	5.06 ± 0.04	4.97 ± 0.04	—	—	—
HbA1c, %							
LPF	5.32 ± 0.01	—	—	5.30 ± 0.01	0.020	0.194	0.253
HPF	5.31 ± 0.01	—	—	5.26^3^ ± 0.01	—	—	—
Triglyceride, mmol/L							
LPF	1.16 ± 0.05	1.27 ± 0.05	1.24 ± 0.05	1.26 ± 0.05	0.566	0.004	0.109
HPF	1.16 ± 0.05	1.08^4^ ± 0.05	1.06^4^ ± 0.05	1.12^4^ ± 0.05	—	—	—
Total cholesterol, mmol/L							
LPF	4.72 ± 0.05	4.54^3^ ± 0.05	4.56 ± 0.05	4.66 ± 0.05	<0.001	0.003	0.005
HPF	4.73 ± 0.05	4.29^3^^,^^4^ ± 0.05	4.40^3^^,^^4^ ± 0.05	4.41^3^^,^^4^ ± 0.05	—	—	—
HDL-C, mmol/L							
LPF	1.44 ± 0.02	1.42 ± 0.02	1.44 ± 0.02	1.46 ± 0.02	0.032	0.235	0.456
HPF	1.45 ± 0.02	1.37^3^ ± 0.02	1.40 ± 0.02	1.42 ± 0.02	—	—	—
LDL-C, mmol/L							
LPF	2.77 ± 0.04	2.60^3^ ± 0.04	2.57^3^ ± 0.04	2.67 ± 0.04	<0.001	0.011	0.005
HPF	2.77 ± 0.04	2.42^3^^,^^4^ ± 0.04	2.51^3^ ± 0.04	2.49^3^^,^^4^ ± 0.04	—	—	—
Ox-LDL, U/L							
LPF	41.9 ± 0.62	—	—	40.2 ± 0.62	<0.001	0.003	0.002
HPF	42.2 ± 0.63	—	—	36.9^3^^,^^4^ ± 0.63	—	—	—
Leptin, ng/mL							
LPF	12.1 ± 0.54	—	—	10.6 ± 0.55	0.060	0.867	0.499
HPF	11.8 ± 0.56	—	—	11.1 ± 0.56	—	—	—
Adiponectin, μg/mL							
LPF	7.87 ± 0.25	—	—	8.13 ± 0.25	0.005	0.016	0.072
HPF	8.00 ± 0.25	—	—	9.17^3^^,^^4^ ± 0.25	—	—	—

1 Values are estimated marginal means ± SEs. Total sample size was *n* = 68 (LPF) and *n* = 65 (HPF). D, day of trial; HbA1c, glycated hemoglobin A1c; HDL-C, HDL cholesterol; HPF, group provided a high-protein, high-fiber supplement shake; LDL-C, LDL cholesterol; LPF, group provided a low-protein, lower-fiber supplement shake; Ox-LDL, oxidized LDL.

2 Data from the per-protocol analysis, where noncompliant participants were removed and data modeled using generalized least-squares regression with time as a within-participants fixed effect, group as a between-participants fixed effect, their interaction, and sex and baseline value (taken at D0) as a covariate.

3 Different from day 0, *P* < 0.05 (Tukey-adjusted within-participant comparison).

4 Different from LPF at that time, *P* < 0.05 (Tukey-adjusted between-group comparison).

### Other outcomes

Questionnaires assessing mood, binge-eating tendencies, and gastrointestinal symptoms, and outcomes including complete blood count, blood chemistry, and immune marker data, are presented in **Supplemental Tables 1–7**. There were no differences observed between groups with respect to mood (Supplemental Table 1), binge-eating behaviors (Supplemental Table 2), or gastrointestinal symptoms (Supplemental Table 3). Descriptive statistics of the BHD can be found in Supplemental Table 4. Minor group differences were identified for complete blood cell counts (Supplemental Table 5) and serum blood chemistry (Supplemental Table 6) but not immune markers (Supplemental Table 7).

## Discussion

This study examined the impact of consuming supplement shake preloads, varying mainly in protein and fiber on body weight, body composition, and metabolic outcomes. The habitual consumption of an HPF preload 30 min before breakfast and lunch resulted in greater weight loss compared with an isocaloric LPF preload in overweight/obese adults. In addition, greater reductions in total cholesterol, LDL cholesterol, and ox-LDL with greater increases in adiponectin concentrations were observed in the HPF group throughout the 84-d randomized controlled trial. These findings support the consumption of a twice-daily high-fiber and high-protein multi-ingredient shake for weight management and improved metabolic health outcomes.

In ITT and PP analyses, both groups exhibited decreases in body weight, with the HPF group losing significantly more weight than the LPF group. Meta-analyses have demonstrated that higher-protein diets are associated with greater weight loss when compared with lower-protein diets (5, 24). Within these studies, the addition of 25–30 g protein/d appears to be sufficient to elicit long-term improvements in weight management (5, 24). The HPF group in this study was assigned an additional 32 g protein/d compared with the LPF group. Additionally, due to dosage and timing limitations of protein absorption, an intake of ∼25–30 g protein at each meal may be ideal for eliciting protein-related health benefits (25). Presently, most Americans consume equal to or greater than this amount at lunch and dinner, although fail to meet this recommendation at breakfast (26). The habitual addition of a high-protein preload before both breakfast and lunch increases daily protein intake, as well as supports the goal of consuming 25–30 g protein at each meal. This may contribute to improved satiety, as a recent meta-analysis showed that higher protein preloads increase fullness ratings more than lower protein preloads (6).

Similarly, a higher intake of fiber, in particular soluble fiber, has been associated with significant reductions in body weight and BMI (14). When taken before a meal, soluble fiber has been associated with slowed rates of consumption, delayed gastric emptying, and increased feelings of satiety (27). Differences in total fiber intake at baseline were observed, in that the HPF group consumed less fiber than the LPF group. Not only did the HPF group consume significantly more fiber across the intervention due to the additional 6 g fiber/d from the HPF preload compared with LPF but, due to this initial imbalance in intake, they saw a larger relative within-group increase in fiber consumption. High fiber intake has been largely associated with weight loss due to its impact on feelings of satiety and satiation (15). Indeed, previous work utilizing the same HPF supplement shake demonstrated reductions in postprandial desire to eat and hunger following shake consumption (18). When taken before a meal, soluble fiber has been associated with slowed rates of consumption and delayed gastric emptying—both mechanisms for subsequent feelings of satiation as well as satiety (28). Through similar mechanisms, higher fiber intake has also been shown to reduce digestible energy, resulting in higher fecal excretion of dietary energy (29, 30). While both groups reported consuming similar energy across the intervention, it is possible there was less absorption of energy in the HPF group, leading to a greater degree of weight loss. However, given the limitations of underreporting intake, reduced accuracy over time, and the tendency to change dietary behaviors during collection days, the inclusion of self-reported food records to assess changes in daily food intake could have contributed to the lack of differences observed within this study (31).

The HPF group demonstrated greater total tissue loss as measured by DXA, while both groups demonstrated decreased fat tissue percentage (in both ITT and PP analyses). The observed loss in total and fat tissue is consistent with expectations of weight loss in a calorie-restricted diet, although it was hypothesized that, with higher consumption of protein, a greater degree of lean mass retention within the HPF group would have been observed. Instead, we observed a small but significant decrease in absolute lean mass in the HPF group that did not occur in the LPF group. However, as a percentage of total tissue, lean mass slightly increased in both groups. Many studies point towards a role of higher protein intake for lean mass preservation during weight loss. Leidy et al. (32) observed greater preservation of lean body mass during a weight-loss trial comparing a high-protein (30% of daily energy) with a normal-protein (15%) diet, with findings the strongest among the participants with obesity. Farnsworth et al. (33) observed similar findings in which women with overweight preserved greater lean mass during weight loss when assigned to a high-protein (27%) over a normal-protein (15%) diet. In the present study, the HPF group consumed 21% of their daily energy from protein, in comparison to 14% in the LPF group. Both groups fell within the AMDR of 10–35% of total energy intake from protein, and both groups consumed protein in quantities greater than the RDA for their age range (34). It is unclear why the HPF group saw a slight decrease in absolute lean mass, but better preservation of lean mass might have occurred if protein intake was closer to the upper limit of the AMDR. Regardless, the small loss observed is not expected to be clinically relevant.

The HPF group demonstrated greater improvements in cardiometabolic outcomes of total cholesterol, total triglycerides, LDL cholesterol, as well as ox-LDL. While weight loss itself has proven to have an independently powerful impact on obesity-related comorbidities (and may potentially explain metabolic improvements in both groups), so has dietary intake. A recent meta-analysis demonstrated that an increase of 1 g soluble fiber/d may produce a change in total cholesterol and LDL cholesterol of –0.045 and –0.057 mmol/L, respectively (35). Mechanistically, these benefits are derived from the binding of soluble fibers with bile acids in the small intestine, resulting in greater excretion of bile, less absorption of bile acids, and ultimately reduced synthesis of serum cholesterol and LDL cholesterol (27). Similar to bile acids, fiber binds and blocks the absorption of glucose in the small intestine, resulting in lower serum glucose concentrations, and less glycation of hemoglobin over time (27), potentially explaining the observed reduction in HbA1c in the HPF group. Given that the fiber in the HPF preload in this study was approximately 40% soluble (2.4 g of 6 g total fiber), it is plausible soluble fiber is a primary mechanism for the beneficial effects observed on cholesterol and HbA1c.

Another potential mechanism for the metabolic benefits observed may lie with the hormone adiponectin. Both groups exhibited increased adiponectin concentrations at D84, with a greater increase observed in the HPF group (+11% vs. +20%). Adiponectin is an endocrine factor that has insulin-sensitizing, antiatherogenic, and anti-inflammatory properties (36). Previous weight-loss trials have demonstrated associations between weight loss and increases in circulating adiponectin concentrations, even in the presence of weight regain (37–39). Dietary fiber intake has also been identified as having an independent impact on raising adiponectin concentrations, even in the absence of weight loss (40). Previous work has observed similar relations with serum adiponectin concentrations linked to lower concentrations of triglyceride, LDL-cholesterol, as well as ox-LDL concentrations (41, 42). Thus, increasing fiber intake alongside weight loss may result in increased circulating adiponectin, which itself may impact (or be a result thereof) the metabolic benefits associated with weight loss.

While this study derives its strength from its rigorous study design and large sample size, it is not without limitations. A drop-out rate of approximately 22–30% was observed between randomization of subjects and completion of the trial. Then, true randomization procedures were used, as opposed to matching or pseudo-randomization, and therefore differences in dietary intake, sex, and weight status occurred. To account for the effects of drop-out and randomization, baseline values and sex were utilized as covariates in all analyses in the PP analysis. While the use of both an ITT and PP analysis may be viewed as a limitation, the primary outcomes were similar in direction between analyses. Furthermore, the PP analysis allowed for the exclusion of noncompliant participants, thus allowing a better understanding of the effect of the preload under optimal conditions. Although compliance and drop-out different between groups, self-reports identified no impact of the HPF formula on mood, binge-eating behavior, or gastrointestinal symptoms. The final sample size in the PP analysis was similar between groups (differing by 3 participants). As with all dietary interventions, it is impossible to balance treatments on all dietary components. While treatments were isocaloric and matched in calorie content, serving size, and volume, the LPF group did have a higher intake of carbohydrates and sugar. Despite this, there was no negative impact on glycemic control, as indicated by no significant difference in fasting insulin, glucose, or HbA1c between groups at D84. Last, the test product (HPF) formula was more complex than simply protein and fiber and included polyphenol-contributing, plant-derived ingredients, prebiotics, probiotics, and vitamins and minerals. We acknowledge that 1 or more of the other components of the preload may have contributed to some of the beneficial effects observed, and future studies could be conducted to understand the potential contribution of these other aspects of the formula.

In summary, drinking a protein- and fiber-based, multi-ingredient supplement shake preload twice daily before breakfast and lunch resulted in greater weight loss and improved metabolic outcomes compared with an isocaloric control with lower protein and fiber. Both treatment groups adhered to an energy-restricted diet for the 84-d intervention, with differences in macronutrient intake across groups but not energy. Thus, diet composition rather than energy reduction alone may influence the success of a weight-loss regimen, potentially including protein and fiber content.

## Supplementary Material

nxac038_Supplemental_File

## Notes

Supported by Beachbody, LLC.

Author disclosures: ELG was an employee of Beachbody, LLC, at the time the work was conducted. MJW is an employee of The Beachbody Company. SAF has ownership in Traverse Science, and CGE was an employee of Traverse Science at the time the work was conducted. HJL is a member of The Beachbody Company Scientific Advisory Board and a member of the Journal's Editorial Board. ME is an employee of KGK Science Inc.

Supplemental Methods and Supplemental Tables 1–7 are available from the “Supplementary data” link in the online posting of the article and from the same link in the online table of contents at https://academic.oup.com/jn/.

Study procedures were executed by an independent clinical research organization (KGK Science, London, ON). Additional study sites overseen by KGK Science included INQUIS Clinical Research (formerly GI Labs, Inc.) and Great Lakes Clinical Trials (of MB Clinical Research and Consulting, LLC)

Abbreviations used: AMDR, Acceptable Macronutrient Distribution Range; BHD, Bowel Habits Diary; C, complement; D, day; HbA1c, glycated hemoglobin; HPF, high protein and fiber (shake); ITT, intent-to-treat; LPF, low protein and lower fiber (control); ox-LDL, oxidized LDL; PP, per-protocol.
