# Supplementary material for: Consuming a Protein and Fiber-Based Supplement Preload Promotes Weight Loss and Alters Metabolic Markers in Overweight Adults in a 12-Week, Randomized, Double-Blind, Placebo-Controlled Trial
Source: J Nutr. 2022 Feb 25;152(6):1415–25. doi: 10.1093/jn/nxac038 (PMC9178960; doi:10.1093/jn/nxac038)
Supplement: nxac038_Supplemental_File [file nxac038_supplemental_file.docx]

Consuming a protein- and fiber-based, nutrient-dense supplement shake preload increases weight loss and alters metabolic markers in overweight adults in a randomized, double-blind, placebo-controlled trial

Glynn E.L. *et al.*

Online Supplementary Material

**Supplementary Methods**

*Full inclusion/exclusion criteria*

Enrollment Criteria:

Participants were enrolled in the study if they met all inclusion and no exclusion criteria.

Inclusion Criteria:

1. Male or female of 25-50 years of age, inclusive.

2. If female, the subject was not of childbearing potential, which was defined as females who have had a hysterectomy or oophorectomy, bilateral tubal ligation or are post-menopausal (natural or surgically with > 1 year since last menstruation)

OR

Females of childbearing potential who agreed to use a medically approved method of birth control and had a negative urine pregnancy test result at screening. Acceptable methods of birth control included:

• Hormonal contraceptives including oral contraceptives, hormone birth control patch (Ortho Evra), vaginal contraceptive ring (NuvaRing), injectable contraceptives (Depo-Provera, Lunelle), or hormone implant (Norplant System) for at least 3 months.

• Double-barrier method

• Non-hormonal IUDs

• Hormonal IUD methods was a stable dose for at least 3 months

• Vasectomy of partner

• Non-heterosexual lifestyle

3. A BMI between 27.0 and < 35.0 kg/m2 (inclusive). Participants met exactly the inclusion BMI at screening and baseline or failed screening. Participants who met the BMI requirement at screening (D-7) with a BMI of < 27.0 or > 35.0 kg/m2 were counseled.

4. Participants that had a stable weight for the past 6 months. Stable weight was defined as not having gained or lost more than 5 kg of body weight throughout the past 6 months (cycling of weight, for example, with increases/decreases less than 5 kg was considered stable).

5. Agreement to comply with dietary recommendations from nutritionists throughout the study that included a 500-kcal energy deficit from their predicted total energy requirements.

6. Agreed to fully comply with all study procedures.

7. Had given voluntary written and informed consent to participate in the study.

8. Determined to be healthy as per laboratory parameters and physical examination.

Exclusion Criteria:

1. Females who were pregnant, breastfeeding, or planning to become pregnant during the study.

2. Subjects who were smokers (tobacco, e-cigarettes) or have been a smoker within the past 1 year from screening.

3. Individuals who carried an epi-pen or those with a diagnosed allergy or who believed that they have an allergy to milk or milk products, crustacean shellfish, tree nuts, plants of the Asteraceae/Compositae/Daisy family, or peanuts.

4. Individuals with a severe allergy to egg, fish, wheat, or soy ingredients as the product was manufactured in a facility that contained these ingredients.

5. The regular use of prescription, over-the-counter health products, or natural health products/dietary supplements being taken for weight loss, cholesterol levels, blood coagulation regulation (e.g. blood thinners, clotting factor replacements, acetylsalicyclic acid, ibuprofen, fish oils, vitamin E), or blood pressure within 4 weeks of screening.

6. Subjects with a history of eating disorders or difficulty swallowing.

7. Current participation or participation within the last 3 months in any weight loss program or diet (for example Weight Watchers, Jenny Craig, DASH diet, Atkins, Mediterranean, etc.).

8. Medical history of thyroid disorders except for subjects diagnosed with hypothyroid and have been on stable medication for at least the last 3 months before enrollment. All subjects with a medical history of hyperthyroid were excluded.

9. Medical history of hypercholesterolemia.

10. Used cholesterol-lowering prescription drugs within the last 6 months.

11. Had Fasting TGs ≥ 200 mg/dL (2.26 mmol/L) or a fasting total cholesterol ≥ 240 mg/dL (6.216 mmol/L). Participants with screening results within 5% of these exclusions were scheduled for Visit 3 within 28 days of screening.

12. Had fasting glucose ≥ 126 mg/dL (≥ 7.0 mmol/L). Participants with screening results within 5% of this exclusion were scheduled for Visit 3 within 28 days of screening.

13. Hypertension defined as untreated systolic blood pressure > 150 mmHg, diastolic blood pressure > 95 mmHg, or the use of prescription high blood pressure/hypertension medications within the last 6 months.

14. Type I or Type II diabetes or used diabetes medication in a preventative setting.

15. Volunteers on antibiotics within 4-weeks of screening were not allowed to “opt-in” to the stool sample subpopulations but could continue to be in the main population of participants in this study.

16. History of gastrointestinal dysfunction or surgery that may influence digestion or absorption.

17. Chronic inflammation or structural abnormality, including a history of digestive tract abnormalities (inflammatory bowel disease, celiac disease, chronic diarrhea, chronic constipation, duodenal or gastric ulcer, gastric retention or obstruction, Gastroesophageal reflux disease (GERD), or symptomatic cholelithiasis).

18. Subjects who had a history of colorectal cancer, bowel resection, rectocele, or colostomy.

19. Known autoimmune disorders.

20. Immunocompromised individuals such as subjects that had undergone organ transplantation, those with rheumatoid arthritis, or subjects diagnosed with human immunodeficiency virus (HIV).

21. Use of medications that inhibit peristaltic movement.

22. Sudden change in bowel movement or failure to defecate following use of a laxative product.

23. Unstable medical conditions that in the opinion of the Qualified Investigator precluded the subject from participating in the study.

24. Anti-anxiety and anti-psychotic medications were assessed by the MD on a case by case basis.

25. Alcohol use > 2 standard alcoholic drinks per day.

26. Alcohol or drug abuse within the last 6 months.

27. Use of medicinal marijuana.

28. Cancer, except skin cancers, completely excised with no chemotherapy or radiation with a follow up that was negative.

29. Subjects with metal fixation plates or screws from a previous surgery.

30. Clinically significant abnormal laboratory results at screening.

31. Participation in a clinical research trial within 30 days before randomization.

32. Allergy or sensitivity to study supplement ingredients.

33. Individuals who were cognitively impaired and/or who were unable to give informed consent.

34. Any other condition which in the Investigator’s opinion could adversely affect the subject’s ability to complete the study or its measures or which could pose a significant risk to the subject.

35. Unwilling or unable to comply with study timeline and procedures.

36. Individuals who did not like the taste of chocolate or shake type drinks.

*Complete beverage ingredients*

HPF beverage:

Ingredients: Protein Blend (Whey protein isolate (milk), Pea protein, Chia (seed), Flax (seed), Quinoa (seed), Sacha inchi (seed)), Cocoa powder (processed with alkali), Pea fiber (seed), Chlorella, Chicory (root extract and root fiber), Rose hips (fruit), Yacon (root), Acerola juice powder (fruit), Astragalus (root), Pomegranate juice powder (fruit), Bilberry (fruit), Blueberry (fruit), Camu‐Camu (fruit), Cordyceps (fungi), Lycium (fruit), Chaga (fungi), MSM (Methylsulfonylmethane), Spinach (leaf), Ashwagandha (root), Kale (Brassica oleracea L. var. acephala) (leaf), Maitake (fungi), Reishi (fungi), Enzyme blend (Amylase, Cellulase, Lactase, Glucoamylase, Alpha‐Galactosidase, Invertase), Bacillus coagulans, Maca (root), Cinnamon (bark), Green tea extract (leaf), Schisandra (fruit), Luo Han Guo extract (fruit), Matcha green tea (leaf), Moringa oleifera (leaf), Organic cane sugar, Natural chocolate flavor, Xanthan gum, Stevia leaf extract, Salt.

LPF beverage:

Ingredients: Maltodextrin, Sugar beet, Natural chocolate flavors, Cocoa powder, Xanthan gum, Stevia, Sodium chloride, Monk fruit, Beta-carotene, Vitamin C, Vitamin D, Vitamin E, Vitamin K2, Vitamin B1, Vitamin B2, Vitamin B6, Folate, Vitamin B12, Biotin, Calcium, Phosphorus, Iodine, Magnesium, Zinc, Chromium, Selenium.

*Profiles of Mood States-2*

The Profiles of Mood States-2 (POMS-2) was administered at all time points. The POMS-2 is a validated self-administered psychological questionnaire that assesses short-term mood states^1^. The questionnaire consists of 65 adjectives describing feelings participants had after consuming the investigational product. All items were scored on a 4-point Likert scale where 0 represented “Not At All” and 4 represented “Extremely,” depicting how they felt at the moment. Total scores represent seven subscales: the tension-anxiety subscale, depression subscale, anger-hostility subscale, fatigue subscale, confusion-bewilderment subscale, vigor-activity subscale, and friendliness subscale. Results are shown in **Supplementary Table 1**.

*Three-Factor Eating Questionnaire*

The Three-Factor Questionnaire (TFEQ) was administered at all time points. The TFEQ is a validated self-administered questionnaire measuring human eating behavior^2^. The questionnaire consists of 18 questions each scored on a 4-point Likert scale where 1 represented “Definitely True” and 4 represented “Definitely False.” Total scores are comprised of three subscales: the restraint of eating subscale, the disinhibition subscale, and the hunger subscale. Results are shown in **Supplementary Table 2**.

*The Binge Eating Scale*

The Binge Eating Scale (BES) was administered at all time points. The BES is a self-administered questionnaire that quantifies the measure of severity of binge eating^3^. The questionnaire is comprised of 16 items each containing three to four statements regarding behavior, thoughts, and emotions. Participants are instructed to indicate which statement in each item best describes how they feel. Each response is scored from 0 (no severity of BES symptoms) to 3 (serious problems on the BES symptoms). Results are shown in **Supplementary Table 2**.

*Modified Gastrointestinal Symptom Rating Scale*

The Modified Gastrointestinal Symptom Rating Scale (GSRS) is a validated, self-administered, scale used for the assessment of gastrointestinal symptoms^4^. The modified GSRS was used to evaluate gastrointestinal health and regularity for healthy participants at all time points. Reflux, Abdominal pain, Indigestion, Diarrhea, and Constipation were five symptoms derived from the combination of the 15 items in the questionnaire (Abdominal pains, Heartburn, Acid reflex, hollow sensations in the stomach, Nausea and vomiting, Borborygmus, Abdominal distension, Eructation, Increased flatus, Constipation, Diarrhea, Loose stools, Hard Stools, Urgent need for defecation, Feeling of incomplete evacuation). All the domains were scored on a 7-point Likert scale where 1 represented “No Discomfort” and 7 represented “Very Severe Discomfort.” Results are shown in **Supplementary Table 3**.

*Bowel Habits Diary and Bristol Stool Scale*

Participants completed the Bowel Habits Diary with Bristol Stool Scale (BSS) throughout the study for 3- days every two weeks. The Bowel Habit Diary consisted of a 6 items questionnaire that enabled quantification of every bowel movement^5^. The BSS contained seven-stool descriptions along with image scales for stool shape and consistency evaluation. The variable of bowel function indicator was determined by stool consistency’s correlation with colonic transit time. Results are shown in **Supplementary Table 4**.

*Complete blood count, blood chemistry, and immune markers*

Complete blood counts (**Supplementary Table 5**) and blood chemistry (**Supplementary Table 6**) were assessed at each time point. Samples were analyzed by LifeLabs (London, On, Canada) using standardized procedures. Seated resting blood pressure and heart rate (**Supplementary Table 6**) were determined from 3 measurements of the same arm obtained at least 1 minute apart. Heart rate was measured using the reading on the automatic blood pressure monitor or, by the clinical coordinator placing their thumb on the subject’s radial artery. While observing a timer, the number of beats was counted for 60 seconds. This was repeated for 3 measurements. Immune markers (IgG, IgA, and IgM) were measured on D0 and D84 (**Supplementary Table 7**).

**Supplementary References**

1. Searight HR, Montone K. Profile of Mood States. In: *Encyclopedia of Personality and Individual Differences*. Springer International Publishing; 2017:1-6. doi:10.1007/978-3-319-28099-8_63-1

2. Stunkard A, Messick S. The Three-Factor Eating Questionnaire to Measure Dietary Restraint, Disinhibition, and Hunger. *J PsychosomolicReseorch*. 1985;29(I):71-83.

3. Escrivá-Martínez T, Galiana L, Rodríguez-Arias M, Baños RM. The binge eating scale: Structural equation competitive models, invariance measurement between sexes, and relationships with food addiction, impulsivity, binge drinking, and body mass index. *Front Psychol*. 2019;10(MAR). doi:10.3389/fpsyg.2019.00530

4. Revicki DA, Wood M, Wiklund I, Crawley J. *Reliability and Validity of the Gastrointestinal Symptom Rating Scale in Patients with Gastroesophageal Reflux Disease*. Vol 7.; 1998.

5. Lewis SJ, Heaton KW. Stool form as a useful guide to intestinal transit time. *Scand J Gastroenterol*. 1997;32(9):920-924.

| **Supplementary Table 1.**Profile of Mood States – 2 Questionnaire of overweight adults that consumed supplement shakes differing in protein and fiber for 12 weeks.^1^ | | | | | | | | |
| --- | --- | --- | --- | --- | --- | --- | --- | --- |
|  |  | **Timepoint** | | | | **P-Value²** | | |
| **Variable** | **Group** | **D0** | **D28** | **D56** | **D84** | **Time** | **Group** | **Time*Group** |
| Total Mood Disturbance | LPF | 51.6 ± 0.8 | 50.5 ± 0.8 | 50.7 ± 0.8 | 49.6 ± 0.8 | 0.009 | 0.425 | 0.987 |
|  | HPF | 51.2 ± 0.8 | 49.6 ± 0.8 | 50.0 ± 0.8 | 49.3 ± 0.8 |  |  |  |
| Anger-Hostility | LPF | 50.9 ± 0.8 | 50.8 ± 0.8 | 50.6 ± 0.8 | 49.0 ± 0.8 | 0.089 | 0.569 | 0.557 |
|  | HPF | 50.4 ± 0.8 | 49.3 ± 0.8 | 50.5 ± 0.8 | 49.6 ± 0.8 |  |  |  |
| Confusion | LPF | 50.7 ± 0.8 | 50.2 ± 0.8 | 51.1 ± 0.8 | 50.7 ± 0.8 | 0.041 | 0.171 | 0.204 |
|  | HPF | 50.7 ± 0.8 | 49.0 ± 0.8 | 50.3 ± 0.8 | 48.4^#^ ± 0.8 |  |  |  |
| Depression | LPF | 51.1 ± 0.8 | 48.9 ± 0.8 | 50.1 ± 0.8 | 49.7 ± 0.8 | 0.218 | 0.517 | 0.560 |
|  | HPF | 50.5 ± 0.8 | 50.4 ± 0.8 | 51.0 ± 0.8 | 50.1 ± 0.8 |  |  |  |
| Fatigue | LPF | 51.6 ± 1.0 | 50.8 ± 1.0 | 50.6 ± 1.0 | 49.8 ± 1.0 | 0.001 | 0.291 | 0.250 |
|  | HPF | 52.8 ± 1.0 | 48.8^#^ ± 1.0 | 48.9^#^ ± 1.0 | 48.1^#^ ± 1.0 |  |  |  |
| Tension | LPF | 52.7 ± 0.8 | 50.0^#^ ± 0.9 | 50.0^#^ ± 0.9 | 49.1^#^ ± 0.9 | <0.001 | 0.479 | 0.562 |
|  | HPF | 51.4 ± 0.9 | 49.3 ± 0.9 | 49.6 ± 0.9 | 49.6 ± 0.9 |  |  |  |
| Vigor | LPF | 49.6 ± 0.8 | 49.0 ± 0.8 | 49.6 ± 0.8 | 50.2 ± 0.8 | 0.512 | 0.503 | 0.822 |
|  | HPF | 49.8 ± 0.8 | 50.1 ± 0.8 | 49.7 ± 0.8 | 50.7 ± 0.8 |  |  |  |
| Friendliness | LPF | 50.4 ± 0.8 | 48.5^*^ ± 0.8 | 48.3 ± 0.8 | 48.5 ± 0.8 | 0.045 | 0.041 | 0.517 |
|  | HPF | 51.1 ± 0.8 | 51.2^*^ ± 0.8 | 50.0 ± 0.8 | 49.5 ± 0.8 |  |  |  |
| ^1^Abbreviations: D, day of trial; LPF, group provided a low protein lower fiber beverage preload, HPF, group provided a high protein high fiber beverage preload. | | | | | | | | |
| ^2^Data were modeled using generalized least squares regression with the same main and interaction effects, with both baseline (taken at D0) and sex as covariates. Values are presented as estimated marginal means ± standard error. *n* = 68 and 65 for the LPF and HPF groups. | | | | | | | | |
| ^*^Different from LPF at that time, P <0.05 (Tukey-adjusted between-group comparison). | | | | | | | | |
| ^#^Different from day 0, *P* < 0.05 (Tukey-adjusted within-subject comparison). | | | | | | | | |

| **Supplementary Table 2.**Three-Factor Eating (TFEQ) and Binge Eating Scale (BES) Questionnaires of overweight adults that consumed supplement shakes differing in protein and fiber for 12 weeks.^1^ | | | | | | | | |
| --- | --- | --- | --- | --- | --- | --- | --- | --- |
|  |  | **Timepoint** | | | | **P-Value²** | | |
| **Variable** | **Group** | **D0** | **D28** | **D56** | **D84** | **Time** | **Group** | **Time*Group** |
| **TFEQ** |  |  |  |  |  |  |  |  |
| Cognitive Restraint | LPF | 46.6 ± 1.6 | 53.8^#^ ± 1.7 | 51.8^#^ ± 1.7 | 53.2^#^ ± 1.7 | <0.001 | 0.234 | 0.186 |
|  | HPF | 46.1 ± 1.7 | 56.5^#^ ± 1.7 | 57.3^#^ ± 1.7 | 55.4^#^ ± 1.7 |  |  |  |
| Uncontrolled Eating | LPF | 42.6 ± 1.4 | 34.1^#^ ± 1.4 | 33.2^#^ ± 1.4 | 31.0^#^ ± 1.4 | <0.001 | 0.831 | 0.978 |
|  | HPF | 42.2 ± 1.4 | 33.5^#^ ± 1.4 | 33.1^#^ ± 1.4 | 31.2^#^ ± 1.4 |  |  |  |
| Emotional Eating | LPF | 48.4 ± 2.2 | 39.7^#^ ± 2.2 | 39.0^#^ ± 2.2 | 38.9^#^ ± 2.2 | <0.001 | 0.364 | 0.892 |
|  | HPF | 47.7 ± 2.2 | 37.8^#^ ± 2.2 | 36.1^#^ ± 2.3 | 35.7^#^ ± 2.3 |  |  |  |
| **BES** |  |  |  |  |  |  |  |  |
| Binge Eating Summative Score | LPF | 11.1 ± 0.5 | 8.2^#^ ± 0.5 | 7.5^#^ ± 0.5 | 7.6^#^ ± 0.5 | <0.001 | 0.765 | 0.880 |
|  | HPF | 10.7 ± 0.5 | 8.3^#^ ± 0.5 | 7.7^#^ ± 0.5 | 7.3^#^ ± 0.5 |  |  |  |
| ^1^Abbreviations: D, day of trial; LPF, group provided a low protein lower fiber beverage preload, HPF, group provided a high protein high fiber beverage preload. | | | | | | | | |
| ^2^Data were modeled using generalized least squares regression with the same main and interaction effects, with both baseline (taken at D0) and sex as covariates. Values are presented as estimated marginal means ± standard error. *n* = 68 and 65 for the LPF and HPF groups. | | | | | | | | |
| ^*^Different from LPF at that time, P <0.05 (Tukey-adjusted between-group comparison). | | | | | | | | |
| ^#^Different from day 0, *P* < 0.05 (Tukey-adjusted within-subject comparison). | | | | | | | | |

| **Supplementary Table 3.**Modified Gastrointestinal Symptoms Rating Scale of overweight adults that consumed supplement shakes differing in protein and fiber for 12 weeks.^1^ | | | | | | | | |
| --- | --- | --- | --- | --- | --- | --- | --- | --- |
|  |  | **Timepoint** | | | | **P-Value²** | | |
| **Subscale** | **Group** | **D0** | **D28** | **D56** | **D84** | **Time** | **Group** | **Time*Group** |
| Total Score | LPF | 1.4 ± 0.05 | 1.5 ± 0.05 | 1.5 ± 0.05 | 1.4 ± 0.05 | 0.062 | 0.570 | 0.362 |
|  | HPF | 1.4 ± 0.05 | 1.5 ± 0.05 | 1.4 ± 0.05 | 1.5 ± 0.05 |  |  |  |
| Abdominal Pain | LPF | 1.7 ± 0.09 | 1.7 ± 0.09 | 1.7 ± 0.09 | 1.5 ± 0.09 | 0.090 | 0.336 | 0.489 |
|  | HPF | 1.8 ± 0.09 | 1.8 ± 0.09 | 1.7 ± 0.09 | 1.7 ± 0.09 |  |  |  |
| Reflux | LPF | 1.2 ± 0.05 | 1.1 ± 0.05 | 1.1 ± 0.05 | 1.2 ± 0.05 | 0.437 | 0.490 | 0.663 |
|  | HPF | 1.1 ± 0.05 | 1.2 ± 0.05 | 1.1 ± 0.05 | 1.3 ± 0.05 |  |  |  |
| Indigestion | LPF | 1.7 ± 0.09 | 1.8 ± 0.09 | 1.9 ± 0.09 | 1.7 ± 0.09 | 0.807 | 0.641 | 0.653 |
|  | HPF | 1.8 ± 0.09 | 1.8 ± 0.09 | 1.8 ± 0.09 | 1.8 ± 0.09 |  |  |  |
| Diarrhea | LPF | 1.3 ± 0.07 | 1.5 ± 0.07 | 1.4 ± 0.07 | 1.3 ± 0.07 | 0.116 | 0.959 | 0.356 |
|  | HPF | 1.3 ± 0.07 | 1.4 ± 0.08 | 1.4 ± 0.08 | 1.5 ± 0.08 |  |  |  |
| Constipation | LPF | 1.3 ± 0.07 | 1.5 ± 0.07 | 1.3 ± 0.07 | 1.3 ± 0.07 | 0.117 | 0.663 | 0.948 |
|  | HPF | 1.4 ± 0.07 | 1.5 ± 0.07 | 1.4 ± 0.07 | 1.3 ± 0.07 |  |  |  |
| ^1^Abbreviations: D, day of trial; LPF, group provided a low protein lower fiber beverage preload, HPF, group provided a high protein high fiber beverage preload. | | | | | | | | |
| ^2^Data were modeled using generalized least squares regression with the same main and interaction effects, with both baseline (taken at D0) and sex as covariates. Values are presented as estimated marginal means ± standard error. *n* = 68 and 65 for the LPF and HPF groups. | | | | | | | | |
| ^*^Different from LPF at that time, P <0.05 (Tukey-adjusted between-group comparison). | | | | | | | | |
| ^#^Different from day 0, *P* < 0.05 (Tukey-adjusted within-subject comparison). | | | | | | | | |

| **Supplementary Table 4.**Bowel habits diary and Bristol stool scale outcomes of overweight adults that consumed supplement shakes differing in protein and fiber for 12 weeks.^1^ | | | | | |
| --- | --- | --- | --- | --- | --- |
|  |  | **Time point^2^** | | | |
| **Variable** | **Group** | **D0** | **D28** | **D56** | **D84** |
| Total movements per day | LPF | 1.5 (1.3) | 1.6 (1.5) | 1.5 (1.3) | 1.5 (1.3) |
|  | HPF | 1.3 (1.3) | 1.4 (1.3) | 1.5 (1.3) | 1.4 (1.2) |
| % of movements requiring strain | LPF | 10 (0.0) | 9.2 (0.0) | 7.9 (0.0) | 5.6 (0.0) |
|  | HPF | 16 (0.0) | 17 (0.0) | 12 (0.0) | 11 (0.0) |
| % of incomplete movements | LPF | 13 (0.0) | 11 (0.0) | 11 (0.0) | 8.8 (0.0) |
|  | HPF | 17 (0.0) | 15 (0.0) | 9.6 (0.0) | 11 (0.0) |
| % of movements where laxatives were used | LPF | 0.5 (0.0) | 0.0 (0.0) | 0.0 (0.0) | 0.2 (0.0) |
|  | HPF | 0.0 (0.0) | 0.6 (0.0) | 0.0 (0.0) | 0.0 (0.0) |
| % of movements with a Bristol Stool Scale of 1 | LPF | 0.9 (0.0) | 1.1 (0.0) | 1.6 (0.0) | 2.0 (0.0) |
|  | HPF | 4.5 (0.0) | 3.7 (0.0) | 3.9 (0.0) | 3.3 (0.0) |
| % of movements with a Bristol stool scale of 2 | LPF | 9.3 (0.0) | 7.3 (0.0) | 8.1 (0.0) | 6.4 (0.0) |
|  | HPF | 6.2 (0.0) | 5.8 (0.0) | 4.6 (0.0) | 4.6 (0.0) |
| % of movements with a Bristol stool scale of 3 | LPF | 19 (0.0) | 16 (9.2) | 17 (3.1) | 19 (0.0) |
|  | HPF | 14 (0.0) | 16 (0.0) | 16 (7.7) | 18 (4.6) |
| % of movements with a Bristol stool scale of 4 | LPF | 50 (50) | 46 (50) | 45 (47) | 49 (55) |
|  | HPF | 50 (50) | 48 (44) | 46 (46) | 47 (50) |
| % of movements with a Bristol stool scale of 5 | LPF | 13 (0.0) | 15 (0.0) | 16 (0.0) | 18 (7.3) |
|  | HPF | 15 (0.0) | 17 (13) | 20 (14) | 17 (11) |
| % of movements with a Bristol stool scale of 6 | LPF | 7.0 (0.0) | 13 (0.0) | 9.2 (0.0) | 5.7 (0.0) |
|  | HPF | 10 (0.0) | 8.8 (0.0) | 8.1 (0.0) | 8.9 (0.0) |
| % of movements with a Bristol stool scale of 7 | LPF | 1.1 (0.0) | 1.7 (0.0) | 0.6 (0.0) | 0.2 (0.0) |
|  | HPF | 0.7 (0.0) | 1.7 (0.0) | 0.7 (0.0) | 1.9 (0.0) |
| ^1^Abbreviations: D, day of trial; LPF, group provided a low protein lower fiber beverage preload; HPF, group provided a high protein high fiber beverage preload. | | | | | |
| ^2^Values represent the mean (median) for each variable. *n* = 68 and 65 for the LPF and HPF groups. | | | | | |
|  | | | | | |

| **Supplementary Table 5.**Serum complete blood count outcomes of overweight adults that consumed supplement shakes differing in protein and fiber for 12 weeks.^1^ | | | | | | | | |
| --- | --- | --- | --- | --- | --- | --- | --- | --- |
|  |  | **Timepoint** | | | | **P-Value²** | | |
| **Variable** | **Group** | **D0** | **D28** | **D56** | **D84** | **Time** | **Group** | **Time*Group** |
| Red blood cell count, n·10^12^/L | LPF | 4.7 ± 0.02 | 4.7 ± 0.02 | 4.7 ± 0.02 | 4.7 ± 0.02 | 0.936 | 0.216 | 0.146 |
|  | HPF | 4.7 ± 0.02 | 4.7 ± 0.02 | 4.7 ± 0.02 | 4.7 ± 0.02 |  |  |  |
| Mean corpuscular volume, fL | LPF | 86.4 ± 0.17 | 86.8 ± 0.17 | 86.5 ± 0.17 | 86.7 ± 0.17 | 0.092 | 0.843 | 0.045 |
|  | HPF | 86.4 ± 0.17 | 86.5 ± 0.17 | 86.9 ± 0.17 | 86.6 ± 0.17 |  |  |  |
| Mean corpuscular hemoglobin, pg | LPF | 28.9 ± 0.06 | 28.9 ± 0.07 | 29.0 ± 0.06 | 29.0 ± 0.06 | 0.422 | 0.741 | 0.923 |
|  | HPF | 28.9 ± 0.06 | 28.9 ± 0.07 | 29.0 ± 0.07 | 28.9 ± 0.07 |  |  |  |
| Mean corpuscular hemoglobin concentration, g/L | LPF | 335 ± 0.79 | 333 ± 0.81 | 335 ± 0.80 | 334 ± 0.80 | 0.190 | 0.449 | 0.106 |
|  | HPF | 336 ± 0.80 | 335 ± 0.81 | 334 ± 0.82 | 335 ± 0.81 |  |  |  |
| Red cell distribution width, % | LPF | 13.7 ± 0.06 | 13.7 ± 0.06 | 13.6 ± 0.06 | 13.4*^#^* ± 0.06 | <0.001 | 0.143 | 0.563 |
|  | HPF | 13.6 ± 0.06 | 13.6 ± 0.06 | 13.5 ± 0.06 | 13.4*^#^* ± 0.06 |  |  |  |
| Hemoglobin, g/L | LPF | 136 ± 0.68 | 135 ± 0.69 | 135^*^ ± 0.68 | 135^*^ ± 0.68 | 0.871 | 0.041 | 0.227 |
|  | HPF | 136 ± 0.69 | 136 ± 0.69 | 137^*^ ± 0.70 | 137^*^ ± 0.69 |  |  |  |
| Hematocrit, L/L | LPF | 0.41 ± 0.002 | 0.41 ± 0.002 | 0.40 ± 0.002 | 0.40 ± 0.002 | 0.797 | 0.128 | 0.094 |
|  | HPF | 0.41 ± 0.002 | 0.41 ± 0.002 | 0.41 ± 0.002 | 0.41 ± 0.002 |  |  |  |
| Platelet count, n·10^9^/L | LPF | 278 ± 2.88 | 274 ± 2.95 | 274 ± 2.93 | 275 ± 2.92 | 0.371 | 0.514 | 0.909 |
|  | HPF | 276 ± 2.95 | 273 ± 2.99 | 271 ± 3.02 | 273 ± 2.99 |  |  |  |
| White blood cell count, n·10^9^/L | LPF | 6.1 ± 0.12 | 6.2^*^ ± 0.12 | 6.3 ± 0.12 | 6.2 ± 0.12 | 0.597 | 0.019 | 0.520 |
|  | HPF | 6.0 ± 0.12 | 5.8^*^ ± 0.12 | 6.0 ± 0.12 | 6.0 ± 0.12 |  |  |  |
| Neutrophil count, n·10^9^/L | LPF | 3.5 ± 0.10 | 3.6^*^ ± 0.10 | 3.7 ± 0.10 | 3.7 ± 0.10 | 0.246 | 0.041 | 0.722 |
|  | HPF | 3.4 ± 0.10 | 3.3^*^ ± 0.10 | 3.5 ± 0.10 | 3.5 ± 0.10 |  |  |  |
| Lymphocyte count, n·10^9^/L | LPF | 1.9 ± 0.04 | 2.0 ± 0.04 | 1.9 ± 0.04 | 1.9 ± 0.04 | 0.775 | 0.125 | 0.347 |
|  | HPF | 1.9 ± 0.04 | 1.9 ± 0.04 | 1.9 ± 0.04 | 1.9 ± 0.04 |  |  |  |
| Monocyte count, n·10^9^/L | LPF | 0.5 ± 0.01 | 0.5^*^ ± 0.01 | 0.5 ± 0.01 | 0.5 ± 0.01 | 0.433 | 0.077 | 0.045 |
|  | HPF | 0.5 ± 0.01 | 0.4^*^ ± 0.01 | 0.5 ± 0.01 | 0.5 ± 0.01 |  |  |  |
| Eosinophil count, n·10^9^/L | LPF | 0.1 ± 0.01 | 0.2 ± 0.01 | 0.1^*^ ± 0.01 | 0.1 ± 0.01 | 0.395 | 0.418 | 0.040 |
|  | HPF | 0.1 ± 0.01 | 0.1 ± 0.01 | 0.2^*^ ± 0.01 | 0.2 ± 0.01 |  |  |  |
| ^1^Abbreviations: D, day of trial; LPF, group provided a low protein lower fiber beverage preload, HPF, group provided a high protein high fiber beverage preload; fL, femtoliter; pg, picogram. | | | | | | | | |
| ^2^Data were modeled using generalized least squares regression with the same main and interaction effects, with both baseline (taken at D0) and sex as covariates. Values are presented as estimated marginal means ± standard error. *n* = 68 and 65 for the LPF and HPF groups. | | | | | | | | |
| ^*^Different from LPF at that time, P <0.05 (Tukey-adjusted between-group comparison). | | | | | | | | |
| ^#^Different from day 0, *P* < 0.05 (Tukey-adjusted within-subject comparison). | | | | | | | | |

| **Supplementary Table 6.**Serum blood chemistry of overweight adults that consumed supplement shakes differing in protein and fiber for 12 weeks.^1^ | | | | | | | | |
| --- | --- | --- | --- | --- | --- | --- | --- | --- |
|  |  | **Timepoint** | | | | **P-Value²** | | |
| **Variable** | **Group** | **D0** | **D28** | **D56** | **D84** | **Time** | **Group** | **Time*Group** |
| Aspartate amino transferase, U/L | LPF | 20.0 ± 1.0 | 22.9 ± 1.0 | 20.7 ± 1.0 | 23.5^#^ ± 1.0 | 0.014 | 0.605 | 0.184 |
|  | HPF | 20.1 ± 1.0 | 22.6 ± 1.0 | 21.7 ± 1.0 | 20.9 ± 1.0 |  |  |  |
| Alanine transaminase, U/L | LPF | 19.9 ± 1.4 | 25.4^#^ ± 1.4 | 21.5 ± 1.5 | 23.3^#^ ± 1.4 | 0.002 | 0.615 | 0.043 |
|  | HPF | 20.5 ± 1.4 | 22.7 ± 1.5 | 22.9 ± 1.5 | 21.7 ± 1.5 |  |  |  |
| Alkaline phosphatase, U/L | LPF | 70.0 ± 0.9 | 69.7 ± 0.9 | 69.7 ± 0.9 | 69.8 ± 0.9 | 0.507 | 0.872 | 0.462 |
|  | HPF | 69.5 ± 0.9 | 68.7 ± 0.9 | 70.1 ± 0.9 | 71.1 ± 0.9 |  |  |  |
| Total bilirubin, μmol/L | LPF | 9.0 ± 0.3 | 8.9 ± 0.3 | 9.0 ± 0.3 | 8.8 ± 0.3 | 0.825 | 0.408 | 0.723 |
|  | HPF | 8.9 ± 0.3 | 9.2 ± 0.3 | 9.4 ± 0.3 | 9.4 ± 0.3 |  |  |  |
| Total protein, g/L | LPF | 71.3 ± 0.3 | 71.3 ± 0.3 | 70.6 ± 0.3 | 70.8 ± 0.3 | 0.842 | 0.988 | 0.130 |
|  | HPF | 71.1 ± 0.3 | 70.7 ± 0.3 | 71.2 ± 0.3 | 71.3 ± 0.3 |  |  |  |
| Globulin, g/L | LPF | 26.8 ± 0.3 | 26.2 ± 0.3 | 26.1 ± 0.3 | 26.1 ± 0.3 | <0.001 | 0.212 | 0.596 |
|  | HPF | 26.7 ± 0.3 | 25.5^#^ ± 0.3 | 25.8^#^ ± 0.3 | 26.0 ± 0.3 |  |  |  |
| Uric acid, μmol/L | LPF | 299 ± 4.1 | 310 ± 4.1 | 306 ± 4.1 | 306 ± 4.1 | 0.015 | 0.554 | 0.751 |
|  | HPF | 299 ± 4.2 | 313 ± 4.2 | 313 ± 4.2 | 305 ± 4.2 |  |  |  |
| Blood urea nitrogen, mmol/L | LPF | 4.3 ± 0.1 | 4.1^*^ ± 0.1 | 4.2^*^ ± 0.1 | 4.3 ± 0.1 | 0.572 | 0.002 | 0.309 |
|  | HPF | 4.4 ± 0.1 | 4.5^*^ ± 0.1 | 4.7^*^ ± 0.1 | 4.5 ± 0.1 |  |  |  |
| Creatinine, μmol/L | LPF | 71.8 ± 0.6 | 73.9^#^ ± 0.6 | 72.6 ± 0.6 | 70.8 ± 0.6 | <0.001 | 0.090 | 0.072 |
|  | HPF | 72.2 ± 0.6 | 72.1 ± 0.6 | 71.7 ± 0.7 | 69.0^#^ ± 0.6 |  |  |  |
| Estimated glomerular filtration rate ml·min^-1^·1.73m^-2^ | LPF | 99.7 ± 0.9 | 95.3^*#^ ± 0.9 | 97.5 ± 0.9 | 99.8^*^ ± 0.9 | <0.001 | 0.019 | 0.007 |
|  | HPF | 98.7 ± 1.0 | 99.5^*^ ± 1.0 | 99.6 ± 1.0 | 103^*#^ ± 1.0 |  |  |  |
| Albumin, g/L | LPF | 44.5 ± 0.2 | 45.1 ± 0.2 | 44.6^*^ ± 0.2 | 44.7 ± 0.2 | 0.006 | 0.142 | 0.037 |
|  | HPF | 44.4 ± 0.2 | 45.2^#^ ± 0.2 | 45.5^*#^ ± 0.2 | 45.2^#^ ± 0.2 |  |  |  |
| ^1^Abbreviations: D, day of trial; U/L, units per liter; LPF, group provided a low protein lower fiber beverage preload, HPF, group provided a high protein high fiber beverage preload. | | | | | | | | |
| ^2^Data were modeled using generalized least squares regression with the same main and interaction effects, with both baseline (taken at D0) and sex as covariates. Values are presented as estimated marginal means ± standard error. *n* = 68 and 65 for the LPF and HPF groups. | | | | | | | | |
| ^*^Different from LPF at that time, P <0.05 (Tukey-adjusted between-group comparison). | | | | | | | | |
| ^#^Different from day 0, *P* < 0.05 (Tukey-adjusted within-subject comparison). | | | | | | | | |

| **Supplementary Table 6.** Serum blood chemistry of overweight adults that consumed supplement shakes differing in protein and fiber for 12 weeks continued.^1^ | | | | | | | | |
| --- | --- | --- | --- | --- | --- | --- | --- | --- |
|  |  | **Timepoint** | | | | **P-Value²** | | |
| **Variable** | **Group** | **D0** | **D28** | **D56** | **D84** | **Time** | **Group** | **Time*Group** |
| Sodium, mmol/L | LPF | 141 ± 0.2 | 141 ± 0.2 | 141 ± 0.2 | 141 ± 0.2 | 0.240 | 0.414 | 0.091 |
|  | HPF | 141 ± 0.2 | 141 ± 0.2 | 141 ± 0.2 | 141 ± 0.2 |  |  |  |
| Potassium, mmol/L | LPF | 4.4 ± 0.03 | 4.5 ± 0.03 | 4.4 ± 0.04 | 4.3 ± 0.03 | 0.014 | 0.712 | 0.740 |
|  | HPF | 4.4 ± 0.04 | 4.5 ± 0.04 | 4.4 ± 0.04 | 4.4 ± 0.04 |  |  |  |
| Chloride, mmol/L | LPF | 102 ± 0.2 | 102 ± 0.2 | 102 ± 0.2 | 102 ± 0.2 | 0.322 | 0.261 | 0.893 |
|  | HPF | 102 ± 0.2 | 102 ± 0.2 | 102 ± 0.2 | 102 ± 0.2 |  |  |  |
| Calcium, mmol/L | LPF | 2.3 ± 0.01 | 2.4 ± 0.01 | 2.3 ± 0.01 | 2.4 ± 0.01 | 0.079 | 0.923 | 0.301 |
|  | HPF | 2.3 ± 0.01 | 2.3 ± 0.01 | 2.4 ± 0.01 | 2.4 ± 0.01 |  |  |  |
| Phosphorus, mmol/L | LPF | 1.1 ± 0.01 | 1.1 ± 0.01 | 1.1 ± 0.01 | 1.1 ± 0.01 | 0.053 | 0.710 | 0.458 |
|  | HPF | 1.1 ± 0.01 | 1.1 ± 0.01 | 1.1 ± 0.01 | 1.1 ± 0.01 |  |  |  |
| Iron, μmol/L | LPF | 16.5 ± 0.6 | 16.2 ± 0.6 | 16.5 ± 0.6 | 17.0 ± 0.6 | 0.486 | 0.333 | 0.950 |
|  | HPF | 17.0 ± 0.6 | 16.5 ± 0.6 | 17.3 ± 0.6 | 17.5 ± 0.6 |  |  |  |
| Systolic blood pressure, mmHg | LPF | 119 ± 0.8 | 117 ± 0.8 | 118 ± 0.8 | 117 ± 0.8 | 0.642 | 0.621 | 0.111 |
|  | HPF | 118 ± 0.8 | 118 ± 0.8 | 118 ± 0.8 | 119 ± 0.8 |  |  |  |
| Diastolic blood pressure, mmHg | LPF | 75.8 ± 0.6 | 75.9 ± 0.6 | 75.5 ± 0.6 | 75.7 ± 0.6 | 0.197 | 0.622 | 0.195 |
|  | HPF | 75.7 ± 0.6 | 74.6 ± 0.6 | 74.9 ± 0.6 | 76.8 ± 0.6 |  |  |  |
| Heart rate, bpm | LPF | 71.3 ± 0.8 | 71.2 ± 0.8 | 72.2 ± 0.8 | 70.1 ± 0.8 | 0.433 | 0.588 | 0.239 |
|  | HPF | 71.0 ± 0.8 | 70.4 ± 0.8 | 70.7 ± 0.8 | 71.1 ± 0.8 |  |  |  |
| ^1^Abbreviations: D, day of trial; LPF, group provided a low protein lower fiber beverage preload, HPF, group provided a high protein high fiber beverage preload. | | | | | | | | |
| ^2^Data were modeled using generalized least squares regression with the same main and interaction effects, with both baseline (taken at D0) and sex as covariates. Values are presented as estimated marginal means ± standard error. *n* = 68 and 65 for the LPF and HPF groups. | | | | | | | | |
| ^*^Different from LPF at that time, P <0.05 (Tukey-adjusted between-group comparison). | | | | | | | | |
| ^#^Different from day 0, *P* < 0.05 (Tukey-adjusted within-subject comparison). | | | | | | | | |

| **Supplementary Table 7.** Serum concentrations of immune proteins in overweight adults that consumed supplement shakes differing in protein and fiber for 12 weeks.^1^ | | | | | | |
| --- | --- | --- | --- | --- | --- | --- |
|  |  | **Timepoint** | | **P-Value²** | | |
| **Variable** | **Group** | **D0** | **D84** | **Time** | **Group** | **Time*Group** |
| Immunoglobulin G, g/L | LPF | 10.5 ± 0.11 | 10.4 ± 0.12 | 0.375 | 0.054 | 0.045 |
|  | HPF | 10.5 ± 0.12 | 10.8^*^ ± 0.12 |  |  |  |
| Immunoglobulin A, g/L | LPF | 2.07 ± 0.06 | 2.06 ± 0.06 | 0.679 | 0.698 | 0.817 |
|  | HPF | 2.10 ± 0.06 | 2.06 ± 0.06 |  |  |  |
| Immunoglobulin M, g/L | LPF | 1.24 ± 0.01 | 1.21 ± 0.01 | 0.727 | 0.093 | 0.056 |
|  | HPF | 1.24 ± 0.01 | 1.25 ± 0.01 |  |  |  |
| Complement component 3, g/L | LPF | 1.36 ± 0.01 | 1.39 ± 0.01 | 0.129 | 0.348 | 0.750 |
|  | HPF | 1.36 ± 0.01 | 1.37 ± 0.01 |  |  |  |
| Complement component 4, g/L | LPF | 0.38 ± 0.13 | 0.38 ± 0.13 | 0.323 | 0.627 | 0.305 |
|  | HPF | 0.56 ± 0.13 | 0.19 ± 0.13 |  |  |  |
| ^1^Abbreviations: D, day of trial; LPF, group provided a low protein lower fiber beverage preload, HPF, group provided a high protein high fiber beverage preload. | | | | | | |
| ^2^Data were modeled using generalized least squares regression with the same main and interaction effects, with both baseline (taken at D0) and sex as covariates. Values are presented as estimated marginal means ± standard error. *n* = 68 and 65 for the LPF and HPF groups. | | | | | | |
| ^*^Different from LPF at that time, P <0.05 (Tukey-adjusted between-group comparison). | | | | | | |
| ^#^Different from day 0, *P* < 0.05 (Tukey-adjusted within-subject comparison). | | | | | | |
